# Exploring Intensity-Dependent Echogenic Response to Percutaneous Electrolysis in Tendon Tissue: A Cadaveric Study

**DOI:** 10.3390/jcm14134772

**Published:** 2025-07-06

**Authors:** Miguel Malo-Urriés, Jacobo Rodríguez-Sanz, Sergio Borrella-Andrés, Isabel Albarova-Corral, Juan Carlos Martínez-Zamorano, Carlos López-de-Celis

**Affiliations:** 1 Department of Physiatry and Nursing, Health Sciences Faculty, University of Zaragoza, 50009 Zaragoza, Spain; malom@unizar.es (M.M.-U.); sergiocai04@gmail.com (S.B.-A.); ialbarova@unizar.es (I.A.-C.); 2 PhysiUZerapy Health Sciences Research Group, University of Zaragoza, 50009 Zaragoza, Spain; 3 Department of Medicine, Faculty of Medicine and Health Sciences, Universitat Internacional de Catalunya Sant Cugat del Vallès, 08195 Barcelona, Spain; zamorano810@hotmail.com; 4 ACTIUM Functional Anatomy Group, Faculty of Medicine and Health Sciences, Universitat Internacional de Catalunya Sant Cugat del Vallès, 08195 Barcelona, Spain; carlesldc@uic.es; 5 Department of Physiotherapy, Faculty of Medicine and Health Sciences, Universitat Internacional de Catalunya Sant Cugat del Vallès, 08195 Barcelona, Spain; 6 Study Group on Pathology of the Locomotor System in Primary Care (GEPALAP), Institut Universitari d’Investigació en Atenció Primària (IDIAP Jordi Gol), 08007 Barcelona, Spain

**Keywords:** percutaneous electrolysis, dosage, quantitative ultrasound, echogenic response, galvanic current, cadaveric tendon

## Abstract

**Background**: Percutaneous electrolysis (PE) is an emerging therapeutic approach for tendinopathies, applying a galvanic current through a dry-needling needle to induce regenerative tissue responses. However, current dosing strategies are often empirical and lack objective physiological feedback. **Objective**: This study aimed to evaluate the echogenic effects of different galvanic current intensities on cadaveric tendon tissue using quantitative ultrasound. **Methods**: An ex vivo study was conducted on 29 cadaveric patellar tendon samples, each exposed to a single intensity (0–10 mA for 1 s). Quantitative ultrasound analysis was performed post-intervention, and echogenic variables were extracted using UZ eDosifier software. A composite variable, Electrolysis_UZ_Dose, was created via multiple regression to capture the overall ultrasound-visible changes. Data were analyzed using correlation, regression models, and dose–range comparisons. **Results**: An intensity-dependent response was observed in key echogenic parameters. Minimal changes occurred at low intensities (0–2 mA), whereas a progressive response emerged between 2 and 6 mA. Beyond 6 mA, a plateau effect suggested either tissue saturation or imaging limitations due to gas-induced acoustic shadowing. The Electrolysis_UZ_Dose variable strongly correlated with applied intensity (R^2^ = 0.732). **Conclusions**: This study suggests an intensity-dependent echogenic effect of PE on tendon tissue in key ultrasound-derived parameters (A_Number, A_Area, A_Perimeter, A_Homogeneity, and A_ASM). However, as this study was conducted under experimental conditions with a single 1 s application per sample, the results should not be extrapolated to clinical practice without further validation.

## 1. Introduction

Percutaneous electrolysis (PE) is an emerging technique in the fields of physiotherapy and regenerative medicine that involves the application of a continuous galvanic current through a dry-needling needle to induce biochemical effects in biological tissues [1]. Its mechanism of action is thought to be based on the electrochemical phenomenon of electrolysis, in which the direct current promotes the breakdown of molecular bonds, the production of reactive species, alterations in local pH, and cellular damage, all of which stimulate a therapeutic inflammatory response [2]. This local tissue disruption is thought to trigger a cascade of biological events that promotes tissue regeneration, making PE particularly useful in the management of musculoskeletal disorders [3]. Within this context, tendons have become one of the primary targets for percutaneous electrolysis due to their frequent involvement in chronic degenerative pathologies. Some studies have suggested that PE may help modulate inflammation and support tendon repair, although the clinical evidence remains limited and heterogeneous, especially in terms of structural and long-term functional outcomes [4,5]. While different modalities of electrolysis exist, including high- and low-intensity protocols, they all share the common goal of initiating regenerative processes in structurally compromised connective tissue, particularly in chronic tendinopathies where a disorganized and fibrotic collagen matrix is prevalent [3].

PE has been used in conditions such as patellar tendinopathy, where some histological changes have been reported following its application [6], although these findings are based on small sample sizes and lack rigorous controls. One of the main challenges in the clinical application of percutaneous electrolysis lies in the determination of an optimal dose tailored to each patient’s condition and the specific characteristics of the affected tissue. Currently, most clinical protocols are based on predefined electrical parameters, such as current intensity (mA) and application time (s), assuming a linear relationship with therapeutic effect. However, this approach does not necessarily reflect the true biochemical response, as it fails to account for the variability in tissue composition, the severity of degeneration, and individual bioelectrical properties [7]. Moreover, the isolated effects of galvanic current intensity have not been systematically studied, and there is currently no solid evidence regarding how different intensities independently influence tissue behavior. In addition, two recent systematic reviews have emphasized the lack of scientific consensus regarding dosage selection for musculoskeletal disorders, highlighting both the variability of parameter settings and the absence of robust comparative studies between different protocols [8,9].

Some advanced electrolysis devices have begun to incorporate real-time estimations of tissue electrical resistance, enabling a more accurate calculation of the delivered electric charge. However, most current applications still rely on standardized treatment protocols. This standardized approach often overlooks the extent and severity of the lesion being treated—for instance, applying the same dosage to a small focal tendinous injury as to a large, chronic degenerative lesion—disregarding the quantity of affected tissue or its biological state. To date, no dosing approach has directly integrated ultrasound-detectable effects as an objective parameter to guide application. Given that galvanic current induces echogenic changes in the tissue, often visible as hyperechoic foci on ultrasound—presumably caused by hydrogen gas microbubbles generated through the electrolytic reaction [2] —ultrasound imaging may serve as a real-time potential imaging-based indicator of the treatment’s physiological impact. In this context, we propose that ultrasound-visible changes following percutaneous electrolysis could provide valuable feedback for tailoring and optimizing the dosage of galvanic current. Such an approach would represent a significant step forward in the individualization of treatment, potentially allowing the biological response of the tissue—rather than fixed electrical parameters—to determine the appropriate dosage for each intervention.

Therefore, the main objective of this study is to evaluate the echogenic effects of different intensities of galvanic current on cadaveric tendinous tissue, using the patellar tendon as an experimental model. By means of quantitative ultrasound analysis, we aim to explore whether different intensities of galvanic current produce distinguishable echogenic changes in tendon tissue.

## 2. Materials and Methods

### 2.1. Study Design

An ex vivo experimental study was conducted on cadaveric tendinous tissue, using the patellar tendon as a model. The aim was to analyze the ultrasonographic effect of percutaneous electrolysis on tendons at different intensities of galvanic current, assessing echogenic changes through quantitative ultrasound image analysis.

This study was approved by the Local Ethics Committee of the Universitat Internacional de Catalunya (CBAS-2023-11). The principles of good scientific practice in the handling of biological specimens were followed, ensuring data reliability and reproducibility.

A total of 29 cadaveric patellar tendon samples were used. Samples were stored at −20 °C and acclimatized at room temperature for 48 h prior to experimentation.

### 2.2. Experimental Protocol

Each sample was randomly assigned 1 of 29 different galvanic current intensities (0.00, 0.10, 0.20, 0.30, 0.40, 0.50, 0.60, 0.70, 0.80, 0.90, 1.00, 1.50, 2.00, 2.50, 3.00, 3.50, 4.00, 4.50, 5.00, 5.50, 6.00, 6.50, 7.00, 7.50, 8.00, 8.50, 9.00, 9.50, and 10.00 mA), applied for one second each.

The patellar tendon was placed in a standardized position, simulating the typical clinical posture for this intervention, with a consistent 15° knee flexion across all samples to ensure homogeneity in image acquisition. The procedure was performed under ultrasound guidance, using a longitudinal in-plane approach with distal-to-proximal needle entry. The galvanic current was applied by a specialist with over 10 years of experience in ultrasound-guided invasive procedures and specific expertise in percutaneous electrolysis techniques. For the current application, 40 mm × 0.30 mm Agupunt needles were used, along with the Bipolar System device (EPTE Ionclinics, Valencia, Spain), designed to deliver galvanic current in a controlled manner.

**Figure 1:**
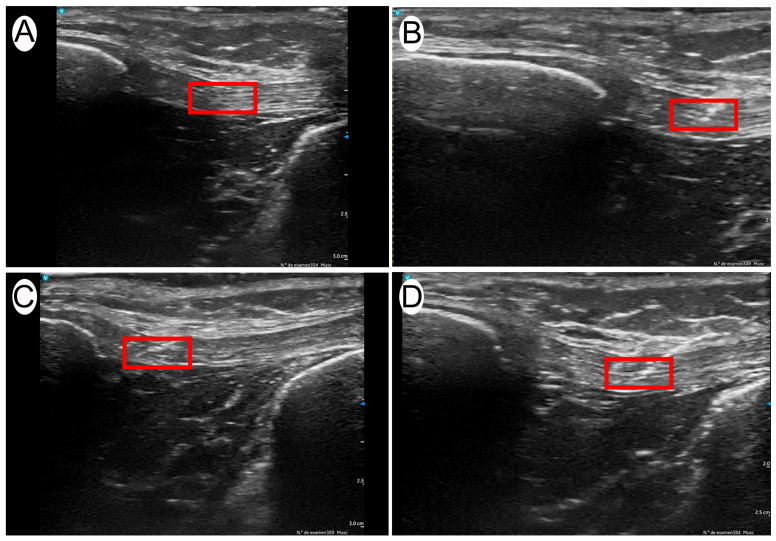
Representative ultrasound images of different percutaneous electrolysis intensities applied for one second: (**A**) 0.1 mA; (**B**) 1 mA; (**C**) 2 mA; (**D**) 6 mA.

**Figure 2:**
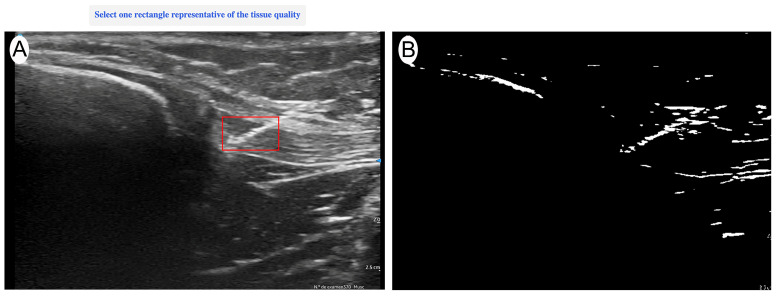
(**A**) Region of interest (ROI) selection. (**B**) Quantitative transformation using UZ eDosifier software to assess the echogenic effects of percutaneous electrolysis.

Ultrasound evaluation was performed with a Vscan portable ultrasound system (General Electric, Boston, MA, USA) immediately after each current application. Imaging was conducted in a longitudinal in-plane view, selecting the best available image that optimally represented the treated area. The evaluation was carried out by an expert with more than 15 years of experience in musculoskeletal (MSK) ultrasound, ensuring an accurate and reproducible assessment of structural changes. The ultrasound evaluator was blinded to the intensity applied to each sample to ensure unbiased image analysis. After the intervention was applied, the needle was temporarily left in place to allow the blinded evaluator to identify the treated area. Once the region of interest (ROI) was manually marked—encompassing the full extent of the needle’s trajectory from the first to the last visible point of the needle within the tendon—the needle was then removed, and quantitative analysis was performed on the selected image (Figure 1). The acquired images were quantitatively analyzed using UZ eDosifier, an adaptation of UZ qTool software [10], specifically developed to assess the echogenic effects of electrolysis on tendon tissue (Figure 2). The development of this tool and its quantitative image-processing workflow have previously been described in detail. This software enables objective image-based measurements, providing information on ultrasonographic characteristics following current application. In this processing step, a standardized global thresholding procedure was applied across all images, using a fixed intensity threshold value of 150 greyscale units, to consistently delineate hyperechoic regions generated by the electrolysis application. This fixed threshold was empirically determined during the software development phase to optimize sensitivity and specificity in detecting gas-related echogenic changes. Table 1 presents the quantified variables. The combination of these measurements enables a quantitative assessment of electrolysis effects on tendon tissue, supporting the identification of response patterns and potential dosage thresholds.

### 2.3. Statistical Analysis

Statistical analysis was performed using IBM SPSS Statistics version 29.0 (IBM, Armonk, NY, USA). A multi-phase approach was implemented to evaluate the quantitative ultrasound changes induced by different intensities of galvanic current applied as percutaneous electrolysis on the patellar tendon.

Initially, an exploratory analysis was conducted using scatter plots to assess the relationship between the applied intensity and each of the measured ultrasound variables. This preliminary analysis enabled the identification of general response patterns, the definition of trends, and the detection of potential thresholds in the tissue’s response to galvanic current. Given the exploratory nature of this study, no single primary outcome variable was predefined. Instead, an initial correlation and regression analysis was performed across all quantified ultrasound parameters to identify those most consistently associated with current intensity. From these, a composite variable (Electrolysis_UZ_Dose) was generated using multiple linear regression to serve as a representative outcome measure, integrating the structural features most sensitive to galvanic current application.

Subsequently, a correlation analysis was performed to examine the association between current intensity and each quantified variable. Spearman’s correlation coefficient was used for this purpose. This analysis identified which variables exhibited a statistically significant association with the applied intensity, providing a preliminary overview of trends in the tissue’s structural changes.

For those variables showing significant correlations with current intensity, a simple linear regression analysis was then carried out. Regression coefficients (B) were calculated to estimate the magnitude of change in each variable as a function of the increasing dose. Coefficients of determination (R^2^), regression coefficients (B), and 95% confidence intervals for the regression coefficients were reported. Additionally, a multiple linear regression model was applied to combine the significant variables into a single optimized summary variable. This predicted variable integrates the influence of multiple ultrasound parameters into a unified metric, allowing for the assessment of the overall impact of galvanic current. Predictor selection was based on statistical significance from the prior analyses. Residuals were analyzed to verify model fit and quality.

## 3. Results

### 3.1. Exploratory Analysis Using Scatter Plots

The exploratory analysis using scatter plots enabled the identification of response patterns between galvanic current intensity and the quantified ultrasound parameters. Some variables showed a clear intensity-dependent response, while others exhibited high dispersion with no apparent trend.

#### 3.1.1. Variables Showing an Apparent Relationship with Current Intensity

**Figure 3:**
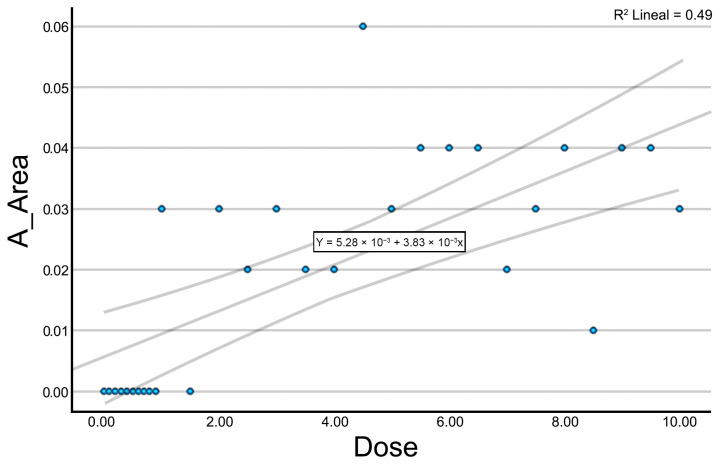
Scatter plot with trend line for the variable A_Area. The trend line is presented solely for exploratory visualization purposes and does not imply the existence of a validated linear relationship. Given the design constraints (single sample per dose and clustered zero values), these plots should be interpreted descriptively rather than inferentially.

A_Number, A_Area, and A_Perimeter demonstrated a positive relationship with the applied intensity. At low intensities (<2 mA), the values remained at zero, whereas a stepwise increase was observed beyond this threshold. At intensities above 6 mA, the values appeared to plateau. Figure 3 displays the results for the A_Area variable. A_Homogeneity and A_ASM exhibited an inverse relationship with current intensity. At low intensities (0–2 mA), values remained constant at 1.00. From 2 mA onwards, a progressive decrease was observed. A_Contrast showed a gradual increase with current intensity, with higher variability observed in the 4–7 mA range and a tendency to stabilize at intensities above 8 mA. B_GLDS_Homogeneity demonstrated a positive relationship with intensity. Although high dispersion was noted at lower intensities, values began to cluster within a more stable range beyond 4 mA.

#### 3.1.2. Variables Without Apparent Relationship with Intensity

In contrast, some variables—such as B_GLCM_Contrast, B_GLCM_SumAverage, B_GLCM_SoSVariance, B_GLCM_DVariance, B_GLCM_Correlation, B_GLCM_IDMoment, B_GLDS_Contrast, B_GLDS_Entropy, B_GLDS_Mean, B_haar_mean, and B_haar_variance—did not show a clear relationship with galvanic current intensity. These variables exhibited high dispersion with no consistent patterns indicative of an intensity–response dependence.

### 3.2. Correlation Analysis

A Spearman correlation analysis was performed between galvanic current intensity and the various quantitative ultrasound parameters (Table 2). The results revealed several statistically significant correlations, suggesting potential relationships between current intensity and the echogenic changes observed in tendinous tissue.

Significant positive correlations (*p* < 0.01) were found between current intensity and the variables A_Number (ρ = 0.817), A_Area (ρ = 0.787), and A_Perimeter (ρ = 0.741) (Table 2). A_Homogeneity (ρ = −0.845, *p* < 0.01) and A_ASM (ρ = −0.743, *p* < 0.01) showed strong negative correlations. A moderate positive correlation was also observed between intensity and A_Contrast (ρ = 0.789, *p* < 0.01). Although a negative correlation was found between intensity and B_GLDS_Entropy (ρ = −0.360, *p* = 0.055), it did not reach conventional statistical significance (*p* < 0.05). In addition, variables such as A_Convexity, B_GLCM_Contrast, and B_haar_variance did not show significant correlations with current intensity (*p* > 0.05).

### 3.3. Simple Linear Regression Analysis

To further assess the relationship between galvanic current intensity and the ultrasound variables, simple linear regression analyses were performed. Table 3 presents the coefficients of determination (R^2^), standard errors of the estimate, regression coefficients (B), and their 95% confidence intervals. A high R^2^ value indicates the proportion of variance in each dependent variable explained by the applied current intensity.

The results revealed significant associations between current intensity and several variables, notably A_Number, A_Area, A_Perimeter, A_Homogeneity, A_Contrast, and A_ASM. Positive relationships were found in variables associated with echogenic changes, while A_Homogeneity and A_ASM showed inverse relationships.

### 3.4. Multiple Linear Regression Analysis

A multiple linear regression analysis was performed to optimize the previous model and integrate the most relevant predictive variables. Based on their statistical significance in the simple regression model, the final predictors included in the model were A_Perimeter, A_Area, A_Number, A_Homogeneity, A_Contrast, and A_ASM. Table 4 presents the coefficients of the multiple regression model. This table summarizes the results of the multiple linear regression, in which the influence of several predictive variables on the dependent variable (applied galvanic current intensity) was evaluated. From this analysis, a new composite variable (Electrolysis_UZ_Dose) was generated, combining information from the most relevant predictors in relation to the applied intensity. The resulting model showed a multiple correlation of R = 0.856, explaining 73.2% of the variance in the applied intensity (R^2^ = 0.732, adjusted R^2^ = 0.659).

**Figure 4:**
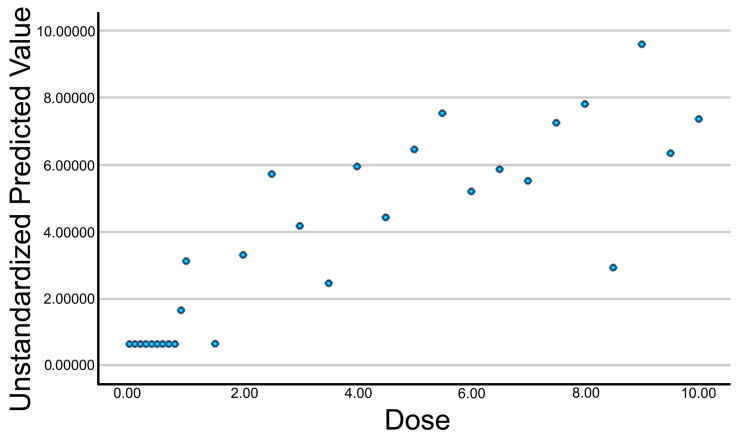
Scatter plot with trend line for the Electrolysis_UZ_Dose variable.

The relationship between the new variable (Electrolysis_UZ_Dose) and the applied galvanic current intensity is shown in Figure 4. A consistent increasing pattern is observed, indicating that the composite variable continues to reflect a clear intensity–response relationship.

## 4. Discussion

The dosage in percutaneous electrolysis (PE) remains a challenge in clinical practice. Currently, the electrical parameters used (current intensity and application time) are often based on empirical criteria, without considering the specific tissue response. This limitation has been previously highlighted in several reviews, where a lack of consensus and robust scientific evidence regarding dosing parameters was identified [8,9]. In this study, we evaluated the effect of different galvanic current intensities on cadaveric tendon tissue using quantitative ultrasound analysis. Our findings suggest that several ultrasound-derived parameters exhibited clear intensity-dependent echogenic variations that may be useful for the exploratory characterization of PE response, although no definitive dose–response relationships could be concluded based on this preliminary data. Importantly, the ultrasound changes quantified in this study are interpreted as the visual manifestation of gas production (mainly hydrogen), resulting from the electrolytic reaction, rather than direct structural remodeling of the tissue.

The intensity of galvanic current applied to tendinous tissue plays a crucial role in modulating the ultrasound-visible effects of percutaneous electrolysis. For exploratory purposes, data were visually grouped into low (0–2 mA), medium (2–6 mA), and high (6–10 mA) ranges to facilitate the descriptive analysis of possible intensity-related patterns. At low intensities (0–2 mA), quantitative ultrasound parameters remained unchanged, suggesting minimal or no echogenic effect. This aligns with previous findings in vivo, where low-intensity protocols failed to induce detectable structural changes, despite equivalent electric charge [6]. In the medium-intensity range (2–6 mA), we identified a consistent and progressive change across several ultrasound-derived parameters, such as A_Number, A_Area, A_Perimeter, A_Homogeneity, and A_ASM. This could indicate the presence of an intensity–response relationship, in line with recent physiological studies suggesting that moderate intensities of galvanic current are sufficient to trigger local inflammation and apoptosis in tendinous tissue, which are necessary for regeneration [3,7]. Above 6 mA, most variables did not show further linear increases. Although values remained elevated compared to lower intensities, no statistically significant differences were found between the medium- and high-intensity groups. This pattern may reflect a ceiling in the visual ultrasound response or a saturation in the measurable effects under our current imaging setup. However, more than reflecting the true saturation of the tissue’s structural response, we hypothesize that this apparent plateau may be partially due to the accumulation of gas generated during electrolysis. As reported in prior studies, electrochemical reactions at the needle tip release sodium hydroxide and hydrogen gas [7,11], which accumulate in the treated area. At higher intensities, this gas formation may cause progressive acoustic shadowing that interferes with the visualization of deeper tissue changes. This phenomenon was clearly observed during the experiment, where posterior artifacts emerged from the subcutaneous layers infiltrated by gas, masking deeper echogenic responses.

In our study, the non-linear dose–response curve we observed—characterized by minimal effects below 2 mA, a clear response between 2 and 6 mA, and a plateau or saturation beyond 6 mA—mirrors recent in vitro findings showing that IL-1β release by macrophages increases up to an optimal electric charge, beyond which cytotoxicity rises and regenerative signaling may diminish [7]. These findings suggest that not only the total dose but also the specific intensity–time configuration determines the biological response, a concept supported by prior reports [2]. Although this is an experimental study, these findings may help explain why, in clinical settings, applying current intensities above a certain threshold (e.g., >2 mA) appears more likely to elicit visible effects, particularly in chronic tendinopathy. However, the clinical efficacy of such intensities remains to be validated in vivo. This may explain why low-intensity protocols, despite delivering the same electric charge, are less effective in promoting structural repair [6]. In summary, our data provide experimental confirmation of what clinical meta-analyses have already suggested but not formally tested: the intensity of galvanic current modulates the ultrasound-detectable impact of electrolysis, and there may exist an optimal physiological range between 2 and 6 mA that maximizes regenerative potential while minimizing adverse effects such as image artifacts or excessive damage [9]. These insights could guide future dose optimization protocols in both research and clinical applications.

To our knowledge, this is the first study to quantify ultrasound-visible changes induced by graded PE intensity using standardized quantitative ultrasound in an ex vivo tendon model. Although several studies have explored clinical outcomes after PE, few have objectively characterized its immediate echogenic impact. Previous reports, such as that of Padrón Benítez and Rojas Mederos, analyzed the structural effects of low- vs. high-intensity PE but did not quantify the ultrasound response in detail [6]. Our results complement those findings by providing objective metrics and supporting the idea that PE dosage generates measurable echogenic effects. However, whether these effects translate into clinically meaningful outcomes remains to be investigated in future studies.

A key aspect of this study was the development of the Electrolysis_UZ_Dose variable, which condenses into a single index the ultrasound-detected effects of galvanic current on tendon tissue. This variable was created through a multiple regression model that integrates the most representative structural parameters, offering a more accurate estimation of tissue response across different intensities. The resulting composite variable showed a consistent upward trend in relation to the applied intensity, suggesting its potential as a quantitative marker of the PE-induced response. Its main advantage lies in combining multiple echogenic features, thereby reducing individual variability and providing a more stable representation of the biochemical current’s effect. To date, previous studies evaluating the clinical effects of percutaneous electrolysis (PE) have primarily relied on subjective outcome measures such as pain and disability scales, and very few have objectively quantified electrolytic changes using imaging. For example, while Minaya-Muñoz et al. reported improvements in epicondylar tendinopathy after PE, they did not demonstrate sonographic morphological changes [12]. In a study by Abat et al., only pre- and post-treatment images from a single patient were presented, without detailed description or quantification of the echographic findings [13]. Similarly, Valera et al. failed to identify significant sonographic changes following six weeks of treatment, although they hypothesized in their discussion that early-stage modifications may have occurred but have been undetectable with the technology available at the time [14]. More recently, semi-quantitative and qualitative sonographic analyses have been used to monitor tendon regeneration in clinical trials. For instance, Padrón Benítez and Rojas Mederos employed standardized musculoskeletal ultrasound to evaluate changes in patellar tendon morphology after high- vs. low-intensity PE. They used objective metrics such as the cross-sectional area at specific locations, echogenicity grading, and neovascularization assessed via power Doppler imaging. Structural changes were only evident in the high-intensity group, reinforcing the idea that the morphological effects of PE depend on intensity thresholds [6]. Similarly, Góngora-Rodríguez et al. demonstrated that improvements in echogenicity, tendon thickness, and vascularity in supraspinatus tendinopathy following combined therapy (PE + peripheral nerve stimulation + eccentric exercise) were correlated and detectable via ultrasound over time [15]. In contrast to these approaches, which rely on qualitative or dichotomous outcomes, the current study introduces a continuous, multidimensional variable capable of capturing subtle intensity-dependent effects of galvanic current. This offers an unprecedented opportunity for dose optimization in both clinical and research settings. Additionally, while prior research has focused mainly on diagnostic or follow-up uses of ultrasound, our method leverages quantitative sonographic texture features as therapeutic monitoring tools. This shift toward objectivity aligns with recent calls for improved standardization and reproducibility in electrotherapy research.

Despite these promising findings, our study presents several limitations. First, the use of cadaveric tissue represents an important limitation, as it does not fully replicate the characteristics of living tissue and obviously does not allow for an assessment of the biological or inflammatory response to PE. Second, it is important to note that the application time (1 s) used in this study does not correspond to standard clinical protocols, where longer durations are common. However, this standardized short duration was essential to isolate and evaluate the effect of current intensity alone, minimizing confounding factors in this ex vivo model. The use of a single evaluator improves internal consistency but limits reproducibility and external validity. Additionally, the application of each intensity level to a single sample precluded intra-sample replication and limited the reproducibility of dose–response observations. Although consistent trends were observed, further studies with repeated applications are needed to validate these results. Future studies should explore the applicability of this methodology to in vivo models and investigate whether ultrasound-based changes correlate with clinical outcomes in patients receiving PE. Although linear regression models were employed to explore the relationship between current intensity and ultrasound-derived variables, we acknowledge that the distribution of some parameters (e.g., A_Area) includes clustered zero values and potential non-normality. This may affect the robustness of the linear fit and limit the generalizability of the regression coefficients. Although these analyses served an exploratory purpose, future studies with larger sample sizes, repeated measurements, and more sophisticated statistical approaches (e.g., non-linear or generalized models) will be essential to validate and better characterize these dose–response relationships. Additionally, the interpretation of a possible “plateau” effect at higher intensities should be considered preliminary, as the current dataset does not offer sufficient statistical power or repeated measurements per dose to confirm such a non-linear trend with confidence. Nonetheless, our primary aim was exploratory, and these analyses were complemented with non-parametric tests (Spearman’s correlation) to confirm the observed trends. Given the exploratory nature of this study, the multiple linear regression model was developed with a limited sample size (n = 29) and included variables with potential collinearity (e.g., A_Area and A_Perimeter). This may reduce the statistical power and robustness of the model, and its findings should be interpreted with caution. Importantly, the composite Electrolysis_UZ_Dose variable proposed here should be considered a conceptual exploratory tool that will require future validation through expanded datasets, dimensionality reduction approaches, and robust statistical modeling. Future studies with larger datasets will be necessary to confirm the validity of the composite variable and apply appropriate collinearity diagnostics. Future studies with larger and more homogeneous datasets, as well as dose-repetition designs, could benefit from applying more advanced or non-linear modeling approaches.

## 5. Conclusions

The application of different intensities of galvanic current to cadaveric patellar tendon tissue produced distinct ultrasound-detectable echogenic effects. Our findings suggest an intensity–response relationship in key parameters such as A_Number, A_Area, A_Perimeter, A_Homogeneity, and A_ASM. Nevertheless, it is important to note that these findings were obtained under experimental conditions with a 1 s application time, and they should not be interpreted as direct clinical recommendations.

## Figures and Tables

**Table 1:** Quantitative ultrasound variables analyzed to evaluate the echogenic effects of percutaneous electrolysis on tendon tissue.

Variable	Description	Interpretation
A_Number	Number of discrete areas detected in the ultrasound image after current application.	Reflects the number of visible marked regions induced by electrolysis.
A_Area	Total area (in cm^2^) of the altered region after the intervention.	Assesses the magnitude of the effect induced by electrolysis.
A_Perimeter	Perimeter (in cm) of the affected region, providing information about the boundaries of tissue alteration.	Indicates the border complexity or irregularity of the echogenic effect induced by electrolysis.
A_Homogeneity:	Measurement of echogenicity uniformity in the image.	A reduction suggests increased heterogeneity in tendon images induced by electrolysis.
A_Contrast	Represents the level of contrast between the treated region and tendon tissue.	An increase indicates greater differentiation in the ultrasound signal induced by electrolysis.
A_ASM (Angular Second Moment)	Texture parameter reflecting pixel organization regularity in the ultrasound image.	A decrease suggests a higher degree of ultrasound signal irregularity induced by electrolysis.
B_GLCM and B_GLDS	B_GLCM (Gray-Level Co-occurrence Matrix): Set of texture features derived from second-order statistical analysis of pixel intensity relationships. Common variables include entropy, dissimilarity, correlation, and variance. B_GLDS (Gray-Level Difference Statistics): Set of texture descriptors based on the absolute differences between gray levels at predefined pixel distances.	Assess different parameters of the complexity of the ultrasonographic image following electrolysis.
B_GLCM_SumAverage: Statistical average of gray levels computed from the GLCM matrix. It provides an overall gray-level intensity trend across the region. B_GLCM_SoSVariance (sum of squares: variance) quantifies the dispersion of gray levels. Higher values indicate greater pixel intensity variability. B_GLCM_DVariance (difference variance): Variance of intensity differences between pixel pairs. It captures local contrast variations in the texture. B_GLCM_Correlation measures the linear dependency of gray levels between neighboring pixels. Higher values reflect more predictable or uniform textures. B_GLCM_IDMoment (inverse difference moment) reflects local homogeneity. Higher values indicate smoother or more homogeneous regions. B_GLDS_Homogeneity measures how similar gray levels are within the region. Higher values indicate more uniform texture. B_GLDS_Contrast captures intensity differences among adjacent pixels. Higher contrast implies more abrupt changes in texture. B_GLDS_ASM (angular second moment) measures energy or uniformity of the texture. Higher values indicate more ordered or regular images. B_GLDS_Entropy quantifies randomness in the image texture. Higher entropy reflects greater complexity. B_GLDS_Mean: Mean gray level difference among neighboring pixels. It represents the average brightness variation within the region. B_haar_mean: Average value derived from Haar wavelet transform applied to the image. It captures broad patterns at multiple scales. B_haar_variance: Variance of coefficients from the Haar transform. It indicates the variability of features across scales.		

**Table 2:** Spearman correlation analysis between galvanic current intensity and evaluated ultrasound parameters.

Variable	Spearman’s Rho	*p*-Value
A_Number	0.817	<0.001
A_Area	0.787	<0.001
A_Perimeter	0.741	<0.001
A_Convexity	−0.111	0.652
A_Homogeneity	−0.845	<0.001
A_Contrast	0.789	<0.001
A_ASM	−0.743	<0.001
B_GLCM_Contrast	−0.073	0.707
B_GLCM_SumAverage	−0.067	0.730
B_GLCM_SoSVariance	0.031	0.873
B_GLCM_DVariance	0.116	0.550
B_GLCM_Correlation	0.039	0.841
B_GLCM_IDMoment	0.179	0.353
B_GLDS_Homogeneity	0.341	0.070
B_GLDS_Contrast	0.179	0.353
B_GLDS_ASM	0.379	0.043
B_GLDS_Entopy	−0.360	0.055
B_GLDS_Mean	0.234	0.222
B_haar_mean	−0.073	0.707
B_haar_variance	0.042	0.829

**Table 3:** Results of linear regression analysis between galvanic current intensity and evaluated ultrasound variables.

Variable	R	R^2^	Standard Error	Beta	B (Dose Coefficient)	IC 95% (Lower/Upper)
A_Number	0.739	0.546	0.944	0.739	0.305	0.195/0.415
A_Area	0.707	0.499	0.013	0.707	0.004	0.002/0.005
A_Perimeter	0.701	0.492	0.363	0.701	0.105	0.063/0.147
A_Homogeneity	0.789	0.623	0.172	−0.789	−0.065	−0.085/−0.045
A_Contrast	0.758	0.574	120.869	0.758	41.398	27.322/55.474
A_ASM	0.674	0.454	0.016	−0.674	−0.004	−0.006/−0.003
B_GLDS_Homogeneity	0.374	0.140	576.264	0.374	68.461	1.350/135.572

**Table 4:** Results of the multiple linear regression analysis.

Variable	B (Coef.)	Standard Error	Beta	CI 95% Lower Bound	CI 95% Upper Bound
Constant	6.038	46.647	—	−90.702	102.777
A_Perimeter	−7.179	3.945	−1.077	−15.36	1.003
A_Area	−2.756	92.578	−0.015	−194.752	189.239
A_Number	0.983	0.636	0.406	−0.337	2.303
A_Homogeneity	−11.32	5.311	−0.937	−22.335	−0.305
A_Contrast	0.012	0.009	0.639	−0.007	0.031
A_ASM	5.922	46.729	0.039	−90.988	102.832

## Data Availability

The data presented in this study are available on request from the corresponding author.
